# Low Prevalence of Adequate eHealth Literacy and Willingness to Use Telemedicine Among Older Adults: Cross-Sectional Study From a Middle-Income Country

**DOI:** 10.2196/65380

**Published:** 2025-07-01

**Authors:** Supawadee Sainimnuan, Rinrada Preedachitkul, Ponnapa Petchthai, Yuwadee Paokantarakorn, Arunotai Siriussawakul, Varalak Srinonprasert

**Affiliations:** 1 Siriraj Health Policy Unit, Faculty of Medicine Siriraj Hospital, Mahidol University, 2 Wanglang Road, Bangkoknoi, Bangkok, 10700, Thailand, 66 2419 7196; 2 Siriraj Geriatric Internal Medicine Research group, Research Department, Faculty of Medicine Siriraj Hospital, Mahidol University, 2 Wanglang Road Bangkok, Bangkok, 10700, Thailand, 66 2419 7196; 3 Integrated Perioperative Geriatric Excellent Research Center, Faculty of Medicine Siriraj Hospital, Mahidol University, Bangkok, Thailand; 4 Department of Anesthesiology, Faculty of Medicine Siriraj Hospital, Mahidol University, Bangkok, Thailand; 5 Division of Geriatric Medicine, Department of Medicine, Faculty of Medicine Siriraj Hospital, Mahidol University, Bangkok, Thailand

**Keywords:** aging population, digital health, digital technology, eHAELS, health information, eHealth literacy scale

## Abstract

**Background:**

Currently, the rapid aging of global population, especially in low- and middle-income countries, is placing changing demands on health care systems. The preparation of the population for adequate eHealth literacy and good digital health is one of the challenges of social policy. The willingness to understand eHealth literacy and telemedicine use across different age groups of the population will help identify loopholes and bottlenecks in the implementation and help to develop appropriate solutions. Currently, studies on the status of eHealth literacy across different age ranges remain limited and scarce.

**Objective:**

In this study, we aimed to investigate the prevalence and factors associated with adequate eHealth literacy, including attitudes toward eHealth literacy and willingness to use telemedicine as an example of digital technology. We focused on the comparison between older people (aged ≥60 years) and younger adult groups in Thailand, a middle-income country.

**Methods:**

We conducted a cross-sectional, observational study from January 2021 to July 2021. A total of 400 participants who visited the outpatient department of Siriraj Hospital were recruited and completed questionnaires collecting demographic information, frequency of internet use, and devices used for accessing the internet. eHealth literacy was assessed using the eHAELS (eHealth Literacy Scale) questionnaire. We also explored the participants’ attitude and willingness to use telemedicine. We applied univariable logistic regression analysis to elucidate the factors associated with eHealth literacy and willingness to use telemedicine.

**Results:**

Our study revealed that the older participants had lower level of eHealth literacy compared to younger participants. Using an eHAELS score ≥26 points to define ‘adequate eHealth literacy,’ 74.0% (n=97) of older adults compared to 22.7% (n=61) of younger adults had inadequate eHealth literacy. Only 19.8% (n=26) of older adults, compared to 65.1% (n=175) of younger adults showed high levels of eHealth literacy defined by exploring each item using the eHEALS tool. The items with the lowest level of eHealth literacy among older adults pertained to confidence in finding and applying health information for self-care and in using information from the internet for making health decisions. In terms of attitude and interest toward telemedicine use, confidence in security, perceived convenience of telemedicine, and adequate eHealth literacy were the three strongest factors associated with willingness to use telemedicine, with odds ratios (ORs) of 5.90 (95% CI 3.43‐10.15), 5.43(95% CI 3.12‐9.43), and 4.45 (95% CI 2.60‐7.62), respectively. Additionally, the younger adults were more likely to be interested in using telemedicine with an OR of 2.02 (95% CI 1.21‐33.37).

**Conclusions:**

Our study addressed the low level of eHealth literacy, with more concerning figures among older adults compared to younger adults in a middle-income country. The willingness to adopt digital technologies related strongly to level of eHealth literacy. This information may be beneficial for guiding further improvements and promoting digital health in low- and middle-income settings facing the challenges of an aging population.

## Introduction

In recent times, digital technologies have dramatically changed the world and become easily accessible. Over the past decade, the widespread adoption of digital technologies has created new opportunities for health care delivery. Digital health tools such as mobile applications, telemedicine platforms, and wearable devices offer cost-effective and scalable methods to improve access to health care services and empower individuals to manage their health more effectively [1]. The shortage of health care facilities, inadequate health infrastructure, and a lack of medical professionals, particularly in remote and underserved areas, are all factors that potentially can be mitigated through implementing digital health tools [1]. The expansion of digital health technologies would be especially beneficial in resource-limited settings, where infrastructure and resources for traditional health care services are often inadequate [1].

During the COVID-19 pandemic, the importance of digital technology has been emphasized in disseminating critical health information in a no-contact society. Health care services around the world rely more on telehealth and telemedicine to deliver health information and medical care [2]. Telehealth and telemedicine play a crucial role in health care, and many studies have reported that they can improve health care access, reduce the cost of care, and enhance the quality of care [3]. However, there are many barriers to its use, including geographical access, availability, affordability, and acceptability [4]. Moreover, the health care systems in many low- and middle-income countries (LMICs) face considerable challenges in providing high-quality, affordable, and universally accessible care through internet-based services.

Demographic change is one of the most significant global trends, where aging populations will be a challenge for health care systems worldwide. The proportion of the older adults is expected to steadily increase at the global level. Given this worrisome change, improving the quality of life among older adults has become one of the crucial issues in public health policy [5]. With the rapid growth of digital technology in health care and related areas, it will inevitably become the core means to deliver various social and health services that can help improve quality of life for the population, particularly for older adults. While older people have more chronic health problems requiring continuous management by various medical professionals, the extent of their ability to access that information through digital technology has not been widely studied [6]. It has been shown that older people, who stand to benefit the most from digital health delivery encountered several difficulties accessing the internet [7]. Moreover, older adults appear to have a high level of anxiety about using smart devices to access health information [8]. These factors may prevent them from gaining benefits from digital technology, which may negatively affect their health and well-being [8].

eHealth literacy refers to the ability to access, understand, and use health information obtained through electronic sources to solve health-related problems [5,9,10]. It reflects skills required to use digital technologies to make decisions regarding health [2]. People with lower eHealth literacy had limited access to technology devices and reduced usage of internet-based resources for health [11,12]. Therefore, they may miss out on opportunities to benefit from several public health interventions delivered through digital tools compared to people with higher eHealth literacy [13]. The study of eHealth literacy and factors associated with low eHealth literacy in people living in LMICs, especially across different age ranges, is essential for developing effective interventions to improve health outcomes and reduce inequalities in the digital world.

It is projected that one-fifth of the world’s population will be > 60 years by 2050, and that 80% of them will reside in LMICs [14]. Thailand, one of the LMICs, is experiencing rapid population aging and has emerged as the second highest aging population in the ASEAN region where the number of older adults will be one-fourth of the population by the year 2030 [15]. The Thai Ministry of Public Health launched strategic plans to promote digital health and implement the use of telemedicine in all hospitals by 2023, with older people as the main targeted population [16,17]. However, studies on the status of eHealth literacy and willingness to use telemedicine in Thailand, particularly among older people, remain limited. This information, especially across different age ranges, will help understand the loopholes and bottlenecks in policy implementation. Therefore, we aim to study level of eHealth literacy compared between older adults and the younger population, and identify the factors associated with attitudes toward eHealth literacy among people of different ages ranges to inform health policy guidelines. Furthermore, the findings of this study may serve as a case study for other LMICs that are also approaching an aging population and aimed to implement digital platforms for older people.

## Methods

### Study Design and Participants

This cross-sectional study was conducted at the outpatient department of Siriraj Hospital, a major medical school that serves as a tertiary referral center in Bangkok, Thailand, from January to July 2021. The inclusion criteria comprised older patients and their relatives visiting the outpatient department. Individuals with communication difficulties such as language or hearing problems, or those who were unable to communicate fluently in Thai, were excluded.

### Measurement Instruments

The questionnaire was divided into three main sections. Part 1 focused on gathering general information about the participants including their age, sex, medical benefit occupation, monthly income, number of doctor visits per year, internet usage, duration of daily internet use, devices used for internet access, and prior experience with telemedicine usage. Part 2 assessed the participants’ eHealth literacy level using the eHealth Literacy Scale (eHEALs). The scale consists of eight questions designed to measure self-perceived ability to use technology for gathering health information combined with the knowledge, perceived comfort, and ability to find, evaluate, and implement electronic health information [18,19]. The scores are obtained by assessing the degree of agreement using a five-point Likert scale ranging from 1 “strongly disagree” to 5 “strongly agree” for each of 8 items. Therefore, the scores could range from 8 to 40, with higher scores indicating higher levels of eHealth literacy [20]. There are several methods for interpreting the results of eHEALs. To categorize eHealth literacy, a cut-off score was used to explore factors associated with overall level of eHealth literacy. For this purpose, we used the eHEALs cut-off score of ≥26 to indicate adequate eHealth literacy [21]. However, another method for classification of eHEALs was also used, by exploring each item of the questionnaire and rating the scores of 4 and 5 per item as ‘agreeing’ [22]. The high level of eHealth literacy was defined as having ‘agreeing’ ≥5 out of 8 eHEALS items [22]. This method would provide more insights into each eHealth literacy issue and may be more meaningful for considering solutions. Part 3 focused on the participants’ attitudes toward accessing telemedicine and included multiple-choice questions and opinion-based questions.

### Data Collection

Research assistants explained the study to the participants using an information sheet. Adequate time was given to the participants to read the information sheet and ask any questions they had before provided written consent. Once consent was obtained, participants were requested to respond to the questionnaire. If any explanations were required, the research assistants provided additional information to help participants complete the questionnaire.

### Ethical Considerations

Ethical approval was obtained from the local ethical board, the Institutional Review Board of Faculty of Medicine, Siriraj Hospital, Mahidol University (SIRB) (COA no. Si 802/2020). Informed consent were obtained from all participants, in accordance with the principle of the Helsinki declaration. Data related to the participants’ identities were not collected in the case record forms. The personal information of participants were anonymized. Participants received US $15 for time expensed in answering the questionnaire.

### Statistical Analysis

The determination of sample size for this study was based on expected differences in proportion of eHealth literacy with the hypothesis that older people would have higher proportions of inadequate eHealth literacy compared to younger population. Inadequate eHealth literacy was classified using eHEALS score <26. Sample size calculation was estimated based on a previous study [21], where participants classified as having inadequate eHealth literacy comprised of 72.8% older participants and the group with adequate eHealth literacy had 57.9% of older people. When considering a two-sided type I error of 0.05 with 80% power, it was determined that 160 participants would be needed in each group. Allowing for 20% of incomplete data, a total of 400 participants were planned for the study.

Demographic information and characteristic variables were summarized using descriptive statistics. Continuous variables were represented by the mean (SD) for normally distributed data or median (IQR) for non-normal distributions. Categorical variables were described using frequency and percentage. For continuous variables, comparisons between the older and younger groups; two-sample *t* tests and Mann-Whitney *U* tests were applied after testing for normality of the data. Pearson’s χ^2^ test or Fisher exact test were used to test categorical variables as appropriate. The analyses were performed using PASW Statistics for Windows (version 29.0, SPSS).

## Results

A total of 400 participants were recruited for the study, including 131 (32.8%) classified as older adults. The demographic information and baseline characteristics are presented in Table 1. Among 131 older adults, 46 (35.1%) were males, and 80 (61.1%) had an education level higher than high school. Most were unemployed (n=88, 67.9%), with an average income of around 10,000 THB (US $285.86) per month and had an average expenditure of 300 THB (US $8.58) per doctor visit. More than half (53.4%) of older adults have never used the internet. Among internet users, the frequency of internet use was around 2 hours per day, with smartphones (n=82, 63.1%) being the most frequently used device for accessing the internet.

Among 269 older adults, 80 (29.7%) were males, and 228 (84.8%) had an education level higher than high school. Most were employed (n =215, 79.9%), with an average income of 20,000 THB (US $571.72) per month, and had an average expenditure of 400 THB (US $11.43) per doctor visit. The majority of younger adults (n=240, 89.2%) regularly used the internet, with an average of 5 hours per day. A total of 254 (94.8%) younger adults used smartphones to access the internet.

**Table 1.** Comparison of demographic information and baseline characteristics of the included population between older persons and younger groups.

Variables	Younger adults’ group (<60 years), (n=269)	Older adults’ group (≥60 years), (n=131)	*P* value
Gender, n (%)			
Male	80 (29.7)	46 (35.1)	.30
Educational level, n (%)			
≥ High school	228 (84.8)	80 (61.1)	<.001
Occupation, n (%)			
Employed	215 (79.9)	43 (32.8)	<.001
Average income (THB/^a^month), median (IQR)	20,000 (10,000-30,000)	10,000 (800-30,000)	<.001
Average cost to see a doctor (THB/time), median (IQR)	400 (200-1000)	300 (150-600)	.06
Experience in using internet, n (%)			
Yes	240 (89.2)	61 (46.6)	<.001
Frequency of internet use^b^ (hr./d), median (IQR)	5 (3-10)	2 (1-3.4)	<.001
Digital devices for using the internet^c^, n (%)			
Computer	45 (16.8)	7 (5.4)	<.001
Notebook	45 (16.8)	8 (6.2)	.004
Tablet	38 (14.2)	9 (6.9)	.046
Smartphone	254 (94.8)	82 (63.1)	<.001

a THB, Thai baht.

b Specified only patient who has access the internet.

c One person may use more than one device.

When comparing the two age groups, the older adult group was more likely to have a lower educational level (*P*<.001), be unemployed (*P*<.001), and have lower income (*P*<.001). They were also less likely to have experience using the internet (*P*<.001) and spent less time online (*P*<.001).

Table 2 presents detailed comparisons of competency in each area of eHealth literacy between the older and younger adult groups. These findings indicate that older adults exhibited significantly lower levels of knowledge in all dimensions of eHealth literacy. Most older adults did not know how to use the internet to find health information for self-care. These findings revealed that less than one-fifth of the older adults knew how to use the internet to find health information and how to use health information available on the internet for self-care. Moreover, less than one-fifth of the older adults were confident in identifying the quality of retrieved information and using it to make health decisions.

Table 3 displays factors associated with eHealth literacy adequacy and attitudes toward telemedicine compared between older and younger adult groups. Approximately one-fourth (n=34) of older adults and 208 (77.3%) of younger adults had adequate eHealth literacy. In both the older and younger adult groups, higher level of education was significantly associated with a level of eHealth literacy. Participants with adequate eHealth literacy are more likely to be interested in telemedicine, leading to a positive attitude and greater confidence in using telemedicine. Nevertheless, a substantial proportion of both the older adult group (n=11, 32.4%) and the younger adult group (n=50, 24.0%) with adequate eHealth literacy remained not confident in using the new technology and declined to use telemedicine. Concerns not directly related to technological competency, such as the adequacy of treatment received through telemedicine and willingness to undergo physical examinations, did not significantly differ between groups with adequate and inadequate eHealth literacy in both age groups. Concerns regarding data security and the adequacy of information received were not significantly different between older adults with adequate and inadequate eHealth literacy.

Factors associated with interest in using telemedicine are demonstrated in Table 4. Lower age, higher education level, higher income, high eHealth literacy, experience in using telemedicine, and belief in the safety and convenience of the service were all significantly associated with willingness to use telemedicine.

**Table 2.** eHealth literacy comparison between the younger and older adults groups.

eHealth literacy variables	Participants with “Agree” (score 4-5)	
	Younger adults group (<60 years), (n=269), n (%)	Older adults group (≥60 years), (n=131), n (%)
I know what health resources are available on the internet.	183 (68.0)	32 (24.4)
I know where to find helpful health resources on the internet.	187 (69.5)	33 (25.2)
I know how to find helpful health resources on the internet.	197 (73.2)	32 (24.4)
I know how to utilize the internet to answer my questions about health.	193 (71.7)	25 (19.1)
I know how to utilize the internet information I find to help me.	185 (68.8)	26 (19.8)
I have the necessary skills to evaluate the health resources I find on the internet.	151 (56.1)	26 (19.8)
I can identify high-quality health resources from low-quality health resources.	135 (50.2)	22 (16.8)
I feel confident in using information from the internet to make my health decisions.	131 (48.7)	22 (16.8)
The high score (4-5) of eHealth literacy in 5 questions and higher.	175 (65.1)	26 (19.8)

**Table 3.** Factors associated with adequacy of eHealth literacy and the willingness to use telemedicine, compared between younger and older adult groups.

Variables	Younger adults group (<60 years) (n=269)			Older adults group (≥60 years), (n=131)		
	Inadequate eHealth literacy (n=61)	Adequate eHealth literacy (n=208)	*P* value	Inadequate eHealth literacy (n=97)	Adequate eHealth literacy (n=34)	*P* value
Living status, n (%)						
Living with family	48 (78.7)	119 (57.2)	.003	69 (71.1)	25 (73.5)	.79
Living alone	13 (21.3)	89 (42.8)		28 (28.9)	9 (26.5)	
Educational level, n (%)						
≥ High school	38 (62.3)	190 (91.3)	<.001	50 (51.5)	30 (88.2)	<.001
Occupation, n (%)						
Employed	50 (82.0)	165 (79.3)	.65	30 (30.9)	13 (38.2)	.44
Frequency of internet use (hr/day)^a^, median (IQR)	2 (1-5)	6 (3-10)	<.001	2 (1-3)	3 (2-15)	.03
Digital devices for using the internet^b^, n (%)						
Computer	4 (6.6)	41 (19.8)	.02	1 (1.0)	6 (17.6)	.001
Notebook	3 (4.9)	42 (20.3)	.003	3 (3.1)	5 (14.7)	.03
Tablet	3 (4.9)	35 (16.9)	.02	4 (4.2)	5 (14.7)	.052
Smartphone	48 (78.7)	206 (99.5)	<.001	49 (51.0)	33 (97.1)	<.001
Attitude of telemedicine, n (%)						
Know or have experience in telemedicine	8 (13.1)	92 (44.2)	<.001	6 (6.2)	17 (50.0)	<.001
Interested in using telemedicine	40 (65.6)	188 (90.4)	<.001	66 (68.0)	30 (88.2)	.02
What would be the reason for declining the use of telemedicine?, n (%)						
Wish to have a physical examination by a doctor	42 (68.9)	120 (57.7)	.12	70 (72.2)	22 (64.7)	.41
Not confident in using new technology	38 (62.3)	50 (24.0)	<.001	64 (66.0)	11 (32.4)	.001
Concerns about data security	20 (32.8)	38 (18.3)	.02	20 (20.6)	5 (14.7)	.45
Concerns of not receiving adequate treatment	32 (52.5)	104 (50.0)	.74	60 (61.9)	15 (44.1)	.07
Concerns of not receiving adequate information	18 (29.5)	25 (12.0)	.001	17 (17.5)	4 (11.8)	.43

a Specified only patient who had access to the internet.

b One person may use more than one device.

**Table 4.** The factors associated with interest in using telemedicine.

Variables	Interested in telemedicine use		Univariate analysis, OR^a^ (95% CI)	*P* value
	Not interested (n=76), n (%)	Interested (n=324), (%)		
Gender				
Male	17 (22.4)	109 (33.6)	1.76 (0.97‐3.16)	.06
Age				
<60 Years	41 (53.9)	228 (70.4)	2.02 (1.21‐3.37)	.007
Education Level				
≥ High school	49 (64.5)	259 (79.9)	2.19 (1.27‐3.77)	.005
Occupation				
Employed	42 (55.3)	216 (66.7)	1.62 (0.97‐2.69)	.06
Average income (THB)^b^				
≥10,000 (≥US $285.86)	38 (65.5)	214 (80.8)	2.20 (1.18‐4.11)	.01
Average Internet us (hr/d), n (%)				
≥5	10 (25.0)	135 (51.3)	3.16 (1.48‐6.73)	.003
eHealth literacy				
Adequate eHealth literacy (eHEALS ≥26)	24 (31.6)	218 (67.3)	4.45 (2.60‐7.62)	<.001
Knowledge of telemedicine	15 (19.7)	108 (33.3)	2.03 (1.10‐3.74)	.02
Confident in security issue				
Very safe	24 (31.6)	237 (73.1)	5.90 (3.43‐10.15)	<.001
Convenient of telemedicine compared to face-to-face service, n (%)				
More convenient	41 (53.9)	280 (86.4)	5.43 (3.12‐9.43)	<.001
Having digital devices for assessing the internet, n (%)	52 (70.3)	287 (88.9)	3.37 (1.83‐6.19)	<.001

a OR: odds ratio.

b THB, Thai baht

Confidence in the safety and convenience of the service, along with adequate eHealth literacy, were the three strongest factors associated with interest in telemedicine, with odds ratios (OR) of 5.90 (95% CI 3.43‐10.15; *P*=.001), 5.43 (95% CI 3.12‐9.43; *P*=.001), and 4.45 (95% CI 2.60‐7.62; *P*=.001), respectively. The younger adults group was more likely than the older adults group to accept telemedicine, with an OR of 2.02 (95% CI 1.21‐3.37; *P*=.007).

## Discussion

### Principal Findings

Our study highlights the very low level of eHealth literacy among older adults compared to younger adults in a middle-income country. Moreover, competency in several aspects of eHealth literacy in both older and younger groups was also unsatisfactory. Several factors associated with a low level of eHealth literacy in this study, such as age, income level, and education level, were nonmodifiable. However, eHealth literacy itself is modifiable through several interventions. The level of eHealth literacy further influences the acceptance and ability to undertake digital health care services. Factors affecting eHealth literacy and attitudes toward accepting telemedicine are key factors to explore and intervene when planning to implement novel digital technologies at the population level.

Several socioeconomic disadvantages are associated with lower levels of eHealth literacy. This study showed results concordant with previous studies [3,23-25], indicating that participants with lower levels of eHealth literacy were more likely to be older and have lower levels of educational attainment. Additionally, low eHealth literacy appeared to be related to inadequate financial resources, resulting in less exposure to digital devices and technologies. This trend was evidenced more prominently among the older adult group in our study, similar to findings from previous studies [23,26].

The concept of eHealth literacy among older individuals has been explored separately [3] from the younger population, as the need for support and barriers to implementation are substantially different. It has been emphasized that eHealth literacy increases with age among young adults, but decreases among older adults [3,23,25-28]. Among older participants in this study, the reported difficulties in eHealth literacy were related to having a low level of knowledge and skills in using the internet. This may also expand their lack of confidence in using information from the internet to make health-related decisions. Several systematic reviews and a recent scoping review demonstrated the benefit of interventions to improve level of eHealth literacy [29-31]. Most studies used health behavior and learning theories. Intervention materials commonly included existing or self-designed websites or applications, with some using incorporating standardized training materials. Outcome measures included information, psychological motivation, and behavioral changes, with only limited studies reporting improved health outcomes. Nevertheless, such strategies should still be considered when planning the delivery policy for implementing digital technologies among older people, not only to include standard materials and tools, but to deliver with more sessions of training programs to enhance confidence for older citizens.

During the COVID-19 pandemic, the importance of digital technologies has skyrocketed for disseminating health information and providing necessary services [22]. Telemedicine was one of the most widely expanded technologies, covering a broader range of health conditions, including preventive, curative, and rehabilitative aspects. The services also expanded to include several chronic medical conditions, including older people with frailty and dementia [32]. Nevertheless, there were some barriers to the adoption of technology among older adults. The top barriers to using telemedicine among older adults were related to a low level of eHealth literacy such as lack of knowledge, difficulty learning, and using the technology [8,21,22,33]. Moreover, concerns related to the privacy and security of the technology, as well as the health outcomes of using online services, were also raised [22]. Our studies discovered similar findings regarding those barriers, which appear to be modifiable through several interventions. However, age-related challenges such as hearing and vision impairments, functional limitations, and ergonomic difficulties, which were not directly addressed in our study, also significantly impact accessibility [22]. For instance, older adults with visual impairments may require larger font sizes, while those with degenerative joint conditions might benefit from specially designed interfaces. Although current conventional digital tools mat not be well-equipped for these changes and prevent older people from using it. These factors were not explored in our study but should also be considered when contemplating plans to implement digital technologies for older people.

Previous studies conducted in high-income countries have shown the benefits of arranging various styles of interventions to improve eHealth literacy in older people [30]. It has been shown that older people can become proficient in using technology, similar to younger adults, with adequate support, understandable explanations of its benefits, and a suitable learning pace [34]. Therefore, encouragement to gain knowledge and skills in digital technologies and having a positive attitude toward the use of telemedicine could enhance uptake in digital health for older adults. Low- and middle income countries are expected to have the highest proportion of older people, along with an expected shortage in several resources for delivering on-site health care services. Using digital health in appropriate service areas would be a promising solution for those settings. Being well prepared by including a training program for older users would increase the chances of successful implementation. Older people have the potential to learn and uptake novel technologies, not less than the younger population, but at different paces. Having volunteers in their communities to provide training through public health centers or activities clubs might be one of the interesting policies to reach more older groups. The process requires considerable work, such as developing local content, coordinating governments and private organizations to provide funding for eHealth literacy projects, and empowering community members to take the initiative to improve their health. However, this will be a sustainable option for the long term. Therefore, to overcome several limitations for older people in resource-limited settings, it is essential to implement a comprehensive policy with ongoing support from public and private sectors to enhance internet accessibility, improve digital literacy and competencies for people in the community. The establishment of a program such as the Intergenerational eHealth Literacy Program with additional support to explore and resolve structural limitations would be beneficial. The Intergenerational eHealth Literacy Program is designed to leverage the technological expertise of younger generations to teach older adults how to search for, understand, and evaluate health information from electronic sources, and apply the knowledge acquired to address health-related issues [35]. Adding a provision program to solve infrastructure issues would be essential. Such comprehensive policy would be an effective strategy to reduce barriers to access to digital technologies and to ensure sustainability.

Our study has some strengths and limitations. Firstly, we used eHAELS questionnaire, a standardized tool that has been validated across several countries with various settings. Secondly, we compared the level of eHealth literacy between older people and younger encounter in the LMIC setting where previous studies mostly conducted from high-income countries and not comparing across age-groups. However, there are several limitations in our studies worth mentioning. Firstly, the study was conducted using cross-sectional design which limits the ability to assess changes in eHealth literary over time. Secondly, the study used self-reported questionnaires to assess eHealth literacy and internet usage which might lead to some reporting bias. Nevertheless, several interventional studies exploring benefits of eHealth literacy and recent systematic reviews demonstrated that eHAELS is the most used and has been widely validated that allowed us to compare across settings [36]. Thirdly, our study was conducted in Thailand, one of the LMIC. The fact that it was conducted in one country, it might not be generalized to other countries with different settings. Fourthly, physical factors affecting eHealth literacy was not explored in this study. Lastly, other psychological aspects, apart from anxiety, such as perceived usefulness; ease of use and complexity of navigation of the sources, might also be affecting the willingness to engage with digital technology. These aspects were not explored in the present study.

### Conclusions

Our study provides insight into the low level of eHealth literacy among older adults compared to younger participants in a middle-income country. We also address the barriers to uptake innovative modes of health care delivery that could be modifiable by providing suitable interventions. Providing information and service through digital technology for older adults would be more successful if adequate support were also prepared.

## Abbreviations

eHEALS: eHealth literacy scale
ETDA: Electronic Transactions Development Agency
LMIC: low- and middle-income country
